# A comprehensive review on the co-occurrence of scurvy and anorexia nervosa

**DOI:** 10.3389/fnut.2024.1466388

**Published:** 2024-09-03

**Authors:** Sunny Cui

**Affiliations:** Department of Biological Sciences, Dartmouth College, Hanover, NH, United States

**Keywords:** vitamin deficiencies, scurvy, anorexia nervosa, eating disorders, vitamin C deficiency, diagnosis and treatment

## Abstract

Scurvy, a rare disease resulting from vitamin C deficiency, can occur in individuals with restrictive eating disorders like anorexia nervosa (AN), leading to severe health complications. This review explores the complex relationship between scurvy and AN, highlighting the overlapping symptoms and challenges in diagnosis and treatment. Vitamin C is essential for collagen synthesis, immune function, and neurotransmitter production, and its deficiency manifests as fatigue, gingival bleeding, joint pain, and perifollicular hemorrhages. AN exacerbates these symptoms through extreme food restriction, causing severe nutritional deficiencies. Analyzing nine case reports, this review reveals that patients with co-occurring AN and scurvy often present with gastrointestinal, psychiatric, and dermatological symptoms. Treatment with vitamin C supplementation typically results in rapid symptom improvement. However, the malnutrition inherent in AN complicates the clinical picture, making timely diagnosis and intervention crucial. This review underscores the importance of a comprehensive, multidisciplinary approach to managing these conditions, emphasizing the need for early recognition and treatment to prevent severe complications. Future research should include a more diverse patient population to enhance understanding of the interplay between AN and scurvy, aiming to improve patient outcomes through tailored treatment strategies.

## 1 Introduction

Scurvy is a rare condition resulting from a deficiency of vitamin C, also known as ascorbic acid. It was first identified in the 18th century by British naval surgeon James Lind, who discovered that citrus fruits could prevent and treat the disease among sailors (1, 2). Vitamin C serves as a cofactor in the biosynthesis of collagen, a structural protein essential for the maintenance and repair of connective tissues, including skin, blood vessels, bones, and cartilage (3). It acts as a reducing agent, donating electrons to specific enzymes that hydroxylate proline and lysine residues in procollagen (3). This hydroxylation allows stabilization and initiation of cross-linking in collagen fibers, providing tensile strength and structural integrity to tissues (3, 4). Therefore, the lack or weakened tensile strength of collagen fibers from vitamin C deficiency explains most of the symptoms observed in patients with scurvy. Moreover, vitamin C has a plethora of other auxiliary functions in the human body. It is involved in the synthesis of neurotransmitters, such as norepinephrine and serotonin, serving a cofactor for the enzymes dopamine β-hydroxylase and tryptophan hydroxylase (1, 3). Furthermore, vitamin C is required for the synthesis of carnitine, a transport molecule of fatty acids into mitochondria for energy production (5). Vitamin C has antioxidant ability, protecting cells from damage by free radicals and regenerating other antioxidants like Vitamin E back to their active forms (2, 4). Finally, Vitamin C plays a role in immune function by stimulating production and function of white blood cells (6).

Early signs of scurvy include fatigue, malaise, and inflammation of the gums, progressing to more severe symptoms if untreated (1). Traditionally, scurvy is characterized by symptoms such as anemia, gingivitis, skin hemorrhages, joint pain, and swelling (1, 2). Other signs include bleeding gums and the loosening of teeth, often accompanied by perifollicular hemorrhages-tiny blood spots around hair follicles (7). These symptoms are among the first indicators that facilitate the diagnosis and subsequent treatment of the disease. Scurvy-induced skin lesions are characterized by the appearance of petechiae, purpura, and bruising, particularly in areas subjected to mechanical pressure such as the legs (7). The skin may become rough and dry, eventually leading to hyperkeratosis and hair follicle abnormalities. If left untreated, the skin will exfoliate, and the condition can become life-threatening. Other symptoms, such as general weakness may also be observed as secondary complications if scurvy persists without intervention. These symptoms result from the impaired function of collagen-dependent tissues and the body’s inability to repair itself adequately (1, 2). The histopathological features of scurvy are often non-specific but can include fragmented collagen fibers, capillary fragility, and minimal inflammatory infiltrate (8). These features contribute to the widespread hemorrhages seen in advanced cases of the disease.

Several factors can cause the nutritional deficiencies that lead to scurvy, including a lack of dietary sources of vitamin C, alcoholism, and medical conditions like gastrointestinal disorders that hinder vitamin absorption (8). Psychological conditions, such as disordered eating, also contribute to insufficient vitamin intake. Anorexia nervosa (AN) exemplifies this, characterized by persistent behaviors that interfere with weight gain, such as severely restricted food consumption (9). This reduced food intake makes AN the mental health disorder with the highest mortality rate, often due to starvation or suicide (10, 11). Diagnosing AN requires a medical professional and adherence to the criteria in the Diagnostic and Statistical Manual of Mental Disorders, 5th Edition (DSM-5) (12). These criteria includes (1) a significant low body weight in the patient’s developmental or biological context due to restricted calorie intake, (2) an intense fear of gaining weight, and (3) having a distorted view of either their body or the medical seriousness associated with low body weight (12). Reduced macronutrient and vitamin intake experienced in patients with AN adversely affects multiple bodily systems. Symptoms in AN patients, including dermatological manifestations, vary based on factors such as purging behavior, illness duration, and overall nutrient intake (13). Common skin-related symptoms include xerosis (dry skin), cheilitis (lip inflammation), acne, effluvium (hair shedding), and nail lesions (13, 14). Other symptoms may include nausea, cachexia (severe weight loss and muscle wasting), constipation, and cardiac issues.

Healthcare providers must understand the overlapping and differential symptoms to differentiate between the two conditions. In scurvy, due to the deficiency of vitamin C, impaired collagen synthesis tends to present as weakened blood vessels, resulting in presentations of petechiae, purpura, and perifollicular hemorrhages, along with characteristic corkscrew hairs and hyperkeratotic papules around hair follicles. Conversely, dermatologic findings in AN are more often related to malnutrition, including xerosis, lanugo hair, and acrocyanosis, with purpura typically only occurring in the context of concomitant thrombocytopenia from severe malnutrition. Gastrointestinal features of AN traditionally include delayed gastric emptying, bloating, and constipation, while symptoms suggestive of defective collagen synthesis such as gum disease or loosening of teeth are more indicative of a scurvy co-diagnosis. Finally, scurvy will tend to present with certain symptoms not traditionally characteristic of AN, such as anemia, musculoskeletal pain from subperiosteal hemorrhages, and “scorbutic rosary” due to costochondral junction enlargement.

Addressing the underlying nutritional deficiencies is crucial for improving patient outcomes and preventing severe complications associated with both disorders. Treatment for scurvy is straightforward and highly effective, typically involving vitamin C supplements or vitamin C-rich foods like citrus fruits, tomatoes, and green leafy vegetables (3). With proper treatment, scurvy symptoms usually resolve rapidly, allowing patients to recover fully. Early diagnosis and intervention are vital to prevent the severe and potentially fatal complications of prolonged vitamin C deficiency.

AN can lead to scurvy due to severe vitamin C deficiencies from restricted food intake, but the relationship is not well understood, with clinical presentations varying widely. While other restrictive eating disorders, such as bulimia nervosa, binge-eating disorder, or avoidant/restrictive food intake, may also raise the risk of scurvy, their pathologies and symptomatic presentations differ significantly from AN due to the different types and amounts of food they consume (15). Therefore, studying these disorders separately is important to understand the specific nutritional deficiencies they cause. This study reviews several case studies from medical literature that document concurrent cases of AN and scurvy, highlighting the symptoms and clinical outcomes of these patients.

## 2 Methods

**Figure 1:**
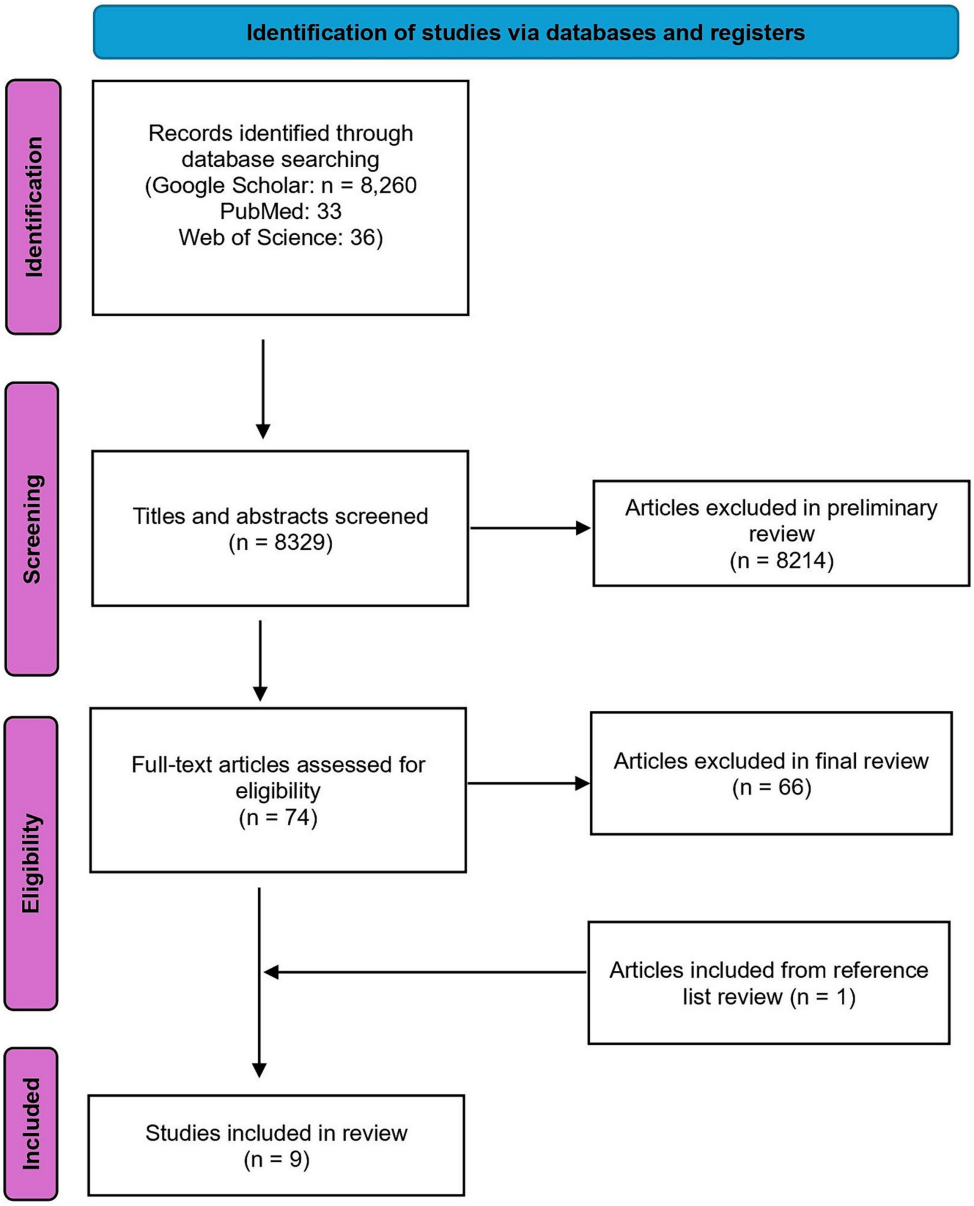
PRISMA diagram.

This study is the most comprehensive literature review to date on the intersection of scurvy and anorexia nervosa (AN). Three major academic databases-Google Scholar, PubMed, and Web of Science-were systematically searched to ensure thorough coverage. The search strategy used a combination of relevant terms: (“scurvy” OR “vitamin C deficiency” OR “ascorbic acid deficiency”) AND “anorexia*,” aimed at capturing all studies related to scurvy and AN, as well as related nutritional deficiencies and their health impacts. The search yielded 8,260 results in Google Scholar, 33 in PubMed, and 36 in Web of Science. Articles available in English and published after 1952 were included to maintain consistency in symptom translation and diagnosis criteria. Abstracts were reviewed to ensure alignment with the research goals, resulting in 52 articles from Google Scholar, 13 from PubMed, and 9 from Web of Science passing the preliminary review, totaling 74 articles. Data extraction used a standardized form to collect information on symptoms, treatment plans, outcomes measured, and key findings related to scurvy and AN. These articles were then thoroughly read in the final review. Articles had to meet specific criteria to pass the final review: reporting a case of AN and scurvy simultaneously, providing original research data or comprehensive reviews, and excluding patients with other medical conditions affecting scurvy or AN diagnosis. Six articles from the preliminary Google Scholar search were included in the final review, along with two new articles from PubMed. No new articles from Web of Science met the inclusion criteria. References of all final review articles were checked to fill any literature gaps, identifying one additional piece of literature. A total of nine case reports were reviewed, with Figure 1 outlining the protocol for literature identification.

## 3 Results

Nine total case reports were analyzed based on the criteria set during the literature search. The total age range of participants was 10–59. Eight reported participants were female, one was male, and three were aged 18 or below at the time of AN and scurvy diagnosis.

Gastrointestinal symptoms were prevalent among the patients. Three case reports documented gingival-related issues: two cases reported gingival bleeding and irritation, one case described gingival hypertrophy. Additionally, decreased bowel sounds and early satiety with epigastric pain and reducing constipation were noted in two other cases. Psychiatric symptoms were also commonly observed. General weakness (asthenia), malaise, or fatigue were reported in six of the nine cases. Cognitive dysfunction was noted in three cases, including delayed speech and motor retardation. Dermatological symptoms were significant and varied across reports. Purpura, rashes, or ecchymosis in the lower extremities was prevalent in every case, while five cases reported perifollicular purpura. Two cases reported corkscrew hairs. Other dermatological symptoms included a hyperpigmented face with dry fissured hands (one case) and hands’ xerosis with nail fragility (one case). Cardiovascular symptoms were less frequently reported. Symptoms included tachycardia (one case), bradycardia with hypotension and poor peripheral perfusion (one case), pulmonary hypertension (one case), and pedal edema (one case). Other symptoms included menorrhagia (one case) and cachexia (two cases). The symptoms recorded for all patients are reported in Table 1.

**Table 1:** Symptoms of patients with concurrent anorexia nervosa and scurvy.

Author	Year	Age, Sex	Gastrointestinal	Psychiatric	Skin	Cardiovascular	Other
da Silva et al. (18)	2023	10 M	Gingival edema and bleeding during tooth brushing	Asthenia (general weakness), weakness, malaise, sadness.	Pain and purpuric lesions on the lower limbs. Swollen and inflamed gums. Symmetrically distributed purpuric and palpable lesions on the lower limbs without edema.	Unreported	Unreported
Du & Kulkarni (19)	2024	15F	Gingival bleeding, Gingival irritation	Fatigue	Rash on both legs, nonblanching perifollicular papules. Mild swelling on her left cheek. Scant cervical lymphadenopathy. Soft, subcutaneous fluid collection on the left knee. Perifollicular hemorrhages. Faint corkscrew hairs. Perifollicular papules. Gingival irritation along the lower gum line.	Tachycardia	Menorrhagia
Gisondi & Bellinato (16)	2022	59F	Unreported	Unreported	Striped purpura and ecchymoses on the legs.	Unreported	Unreported
Levavasseur et al. (33)	2015	38F	Unreported	Unreported	Purpura and spontaneous hematomas on the lower extremities. Perifollicular hyperkeratosis. Hair dystrophy with corkscrew hair. Spontaneous hematomas on the legs.	Unreported	Unreported
Martin-Benllock et al. (20)	2023	37F	Gingival hypertrophy without bleeding. Arthromyalgia in the lower limbs	Anxiety, mood lability. Asthenia. Cognitive schemas and behavioral responses indicative of a dysfunctional personality. Repeated anxiety crises.	Spontaneous ecchymoses. Folliculitis with perifollicular purpura in the lower limbs.	Unreported	Unreported
Peixoto et al. (17)	2018	21F	Unreported	Fatigue, weakness, and myalgias	Ecchymosis. Scattered petechiae on the torso and legs. Hands’ xerosis. Nail fragility. Lanugo-like body hair on arms and back.	Pedal edema	Unreported
Roy-Lavalee et al. (21)	2020	16F	Decreased bowel sounds	Delayed speech and cognitive slowing. Diffuse muscle weakness.	Multiple tiny hyperpigmented perifollicular petechial papules. Corkscrew hairs on the abdomen. Rash on lower leg	Bradycardia. Poor peripheral perfusion. Hypotension. Decreased peripheral pulses.	Cachexia, Unable to ambulate independently
Christopher et al. (22)	2002	46F	Early satiety. Epigastric pain and tenderness. Constipation. Petechiae on hard palate. Poor oral hygiene. Periodontal disease. Temporal wasting. Scant occult blood on rectal examination.	Fatigue, lethargy. Psycho motor retardation.	Nonpruritic rash on lower extremities. Palpable perifollicular papules. Nonpalpable purpuric macules and papules. Hemorrhages and ecchymoses around the knee. Lanugo over the arms.	Easy bruising.	Cachexia
Mehta et al. (23)	1996	40F	Unreported	Weakness, fatigue, myalgia.	Purpuric rash. Hyperpigmented face. Dry fissured skin on hands. Perifollicular purpura. Ecchymosis on the right inner thigh. Focal dermal hemorrhage. Anergy. Perifollicular purpura.	Severe pulmonary hypertension. Dilated pulmonary artery. Ventricular and atrial enlargement on the right side of the heart.	Shortness of breath. Moderate synovial swelling, warmth, and pain on palpation of the ankles. Interstitial edema. Hematuria.

**Figure 2:**
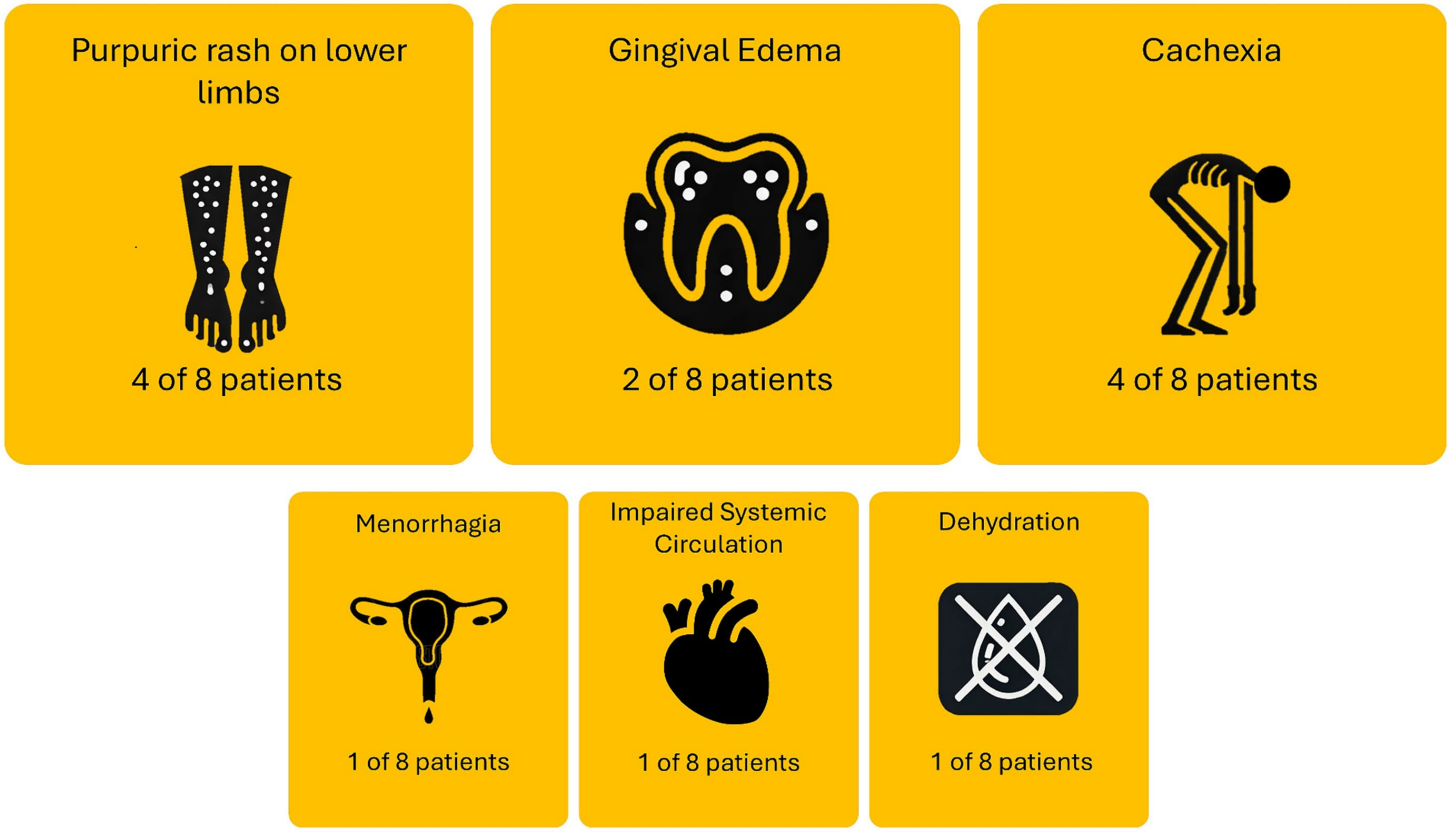
Causes of admission for patients comorbid with scurvy and anorexia nervosa.

The causes of admission of patients comorbid with scurvy and AN are summarized in Figure 2. Four of the eight patients were admitted either solely or partially due to purpura or rashes on the lower limbs. Four of the eight patients were also admitted either solely or partially due to cachexia symptoms. Other reasons patients were admitted include menorrhagia (one case), impaired systemic circulation with poor peripheral perfusion (one case), and dehydration (one case). One patient [59F, recorded by Gisondi and Bellinato (16)] was not included as no description was given on admission reason. One patient [21F, recorded by Peixoto et al. (17)] was admitted due to failing to respond to outpatient care, but no details were provided on the reason for original admittance into outpatient care.

Following diagnosis of scurvy and AN, eight out of eight patients were treated with vitamin C supplementation. One patient (59F, recorded by Gisondi & Bellinato) (16) was not included as treatment regimen and patient outcome was not described. 5 of the 9 cases measured ascorbic acid level prior to treatment, with serum vitamin levels ranging from 0.06 mg/dL - 0.23 mg/dL (RV = 0.4–1.5 mg/dL). BMI ranged between 11.4–17.7 kg/m2. All patients saw either substantial improvements in their symptoms or a complete recovery between 4 and 30 days after initiation of vitamin C supplementation. All patients were given vitamin C orally, at a dosage ranging from 250 mg - 1000 mg daily. Oral vitamin C supplementation was usually sufficient in resolving any dermatological issues and most characteristic symptoms related to scurvy. Dietetic advice, additional vitamin supplementation, or increased caloric intake were used to address other issues. No cases reported the results of subsequent treatment on AN status. The cause for admission, treatment, and outcome results are reported in Table 2.

**Table 2:** BMI, cause of admission, vitamin C level, comorbidities, treatment, and outcome of patients with scurvy and anorexia nervosa.

Author	Weight, Height, BMI	Cause of Admission	Vitamin C Serum (RV: 0.4–2.0 mg/dL)	Comorbidities	Treatment	Outcome
da Silva et al. (18)	27.7 kg, 138 cm, 14.57 kg/m^2^	Pain and purpuric lesions on lower limbs, gingival edema	Ascorbic acid was not measured	Healthy, no comorbidities	100 mg of oral vitamin C thrice daily for 1 week, followed by 100 mg of oral vitamin C once daily	7 days after treatment start, substantial recovery with improved diet, mood, and lower limb pain.
Du & Kulkarni (19)	N/A	Progressive and spreading rash on lower limbs, gingival edema, menorrhagia	<0.09 mg/dL (measured 4 days after discharge)	Not reported	Oral vitamin C supplements, dosage not specified	N/A
Gisondi & Bellinato (16)	N/A	N/A	<0.1 mg/dL	Not Reported	Not Reported	N/A
Levavasseur et al. (33)	N/A	Purpura and spontaneous hematomas on lower limbs	Ascorbic acid was not measured	Chronic Hepatitis C, Iron Deficiency, history of drug addiction	1 g of oral vitamin C daily for 1 month	1 month after treatment start for complete recovery.
Martin-Benllock et al. (20)	42.5 kg, 167 cm,15.24 kg/m^2^	Cachexia, severe malnutrition	0.06 mg/dL	Anxiety, no DSM diagnosis	1 g of oral vitamin C daily for 1 month. 100 mg of oral iron daily and 5 mg of folate daily.	4 days after treatment start, substantial improvement of joints, skin, and gait.
Peixoto et al. (17)	N/A, N/A, 11.4 kg/m^2^	Failure to respond to outpatient treatment	Ascorbic acid was not measured	Not reported	300 mg of oral vitamin C daily for 6 weeks	14 days after treatment start, improved cutaneous signs
Roy-Lavalee et al. (21)	33.1 kg, 170.2 cm, 11.43 kg/m^2^	Cachexia, low weight and cardiovascular support for bradycardia, poor peripheral perfusion, hypotension, dehydration	0.23 mg/ dl	Healthy, no comorbidities	250 mg of oral vitamin C daily. Paediatric multivitamin that included 50 mg of sodium ascorbate daily.	4 weeks after treatment start, improved cutaneous signs
Christopher et al. (22)	38.6 kg, 165 cm, 14.18 kg/m^2^	Cachexia	Ascorbic acid was not measured	Nephrolithiasis, malnutrition, and normocytic anemia. Surgical history included hysterectonomy, bilateral salpingo-oophorectomy,	Treated with ascorbic therapy via vitamin supplements and enteral supplements. Did not specify duration.	7 days after treatment start, complete recovery.
Mehta et al. (23)	44 kg, 157.5 cm, 17.74 kg/m^2^	Cachexia (myalgia, weakness, fatigue) and purpura in lower limbs	0.2 mg/dL	Amenorrheic since birth	supplemental vitamin C and other supportive therapies. Did not specify duration.	12 days after treatment start, substantial recovery.

## 4 Discussion

The symptoms experienced by patients with co-occurring AN and scurvy highlight the multifaceted impact of these conditions. Common symptoms such as fatigue and weakness were observed in seven cases (17–23) while purpuric lesions or petechiae were noted in all nine cases. These findings emphasize that fatigue and weakness are prevalent in both scurvy and AN patients, with skin manifestations like purpuric lesions and petechiae being particularly common in scurvy but likely exacerbated by malnutrition from AN. The prevalence of psychiatric symptoms, including asthenia and cognitive dysfunction, are also compounded by the psychological stressors inherent in AN. Research indicates that both AN and scurvy can impair cognitive function through mechanisms involving malnutrition and vitamin deficiency-induced neural damage (24–27). To address these complex symptoms, a comprehensive treatment approach is likely beneficial. This should include administering vitamin C and other necessary vitamins that are deficient in these cases to alleviate symptoms of scurvy and related deficiencies, while also gradually increasing dietary intake from the onset to address all nutritional deficiencies associated with AN. Given the significant psychiatric manifestations, ongoing psychiatric evaluation and support should be integrated into the treatment regimen, helping manage immediate symptoms and prevent behavioral relapse. Additionally, patients presented with dermatological manifestations in this study such as a hyperpigmented face, dry fissured hands, and nail fragility suggest that the combination of AN and scurvy may lead to complex skin presentations, as corroborated by previous case studies documenting similar symptoms in isolated cases of scurvy and AN (8, 9).

**Figure 3:**
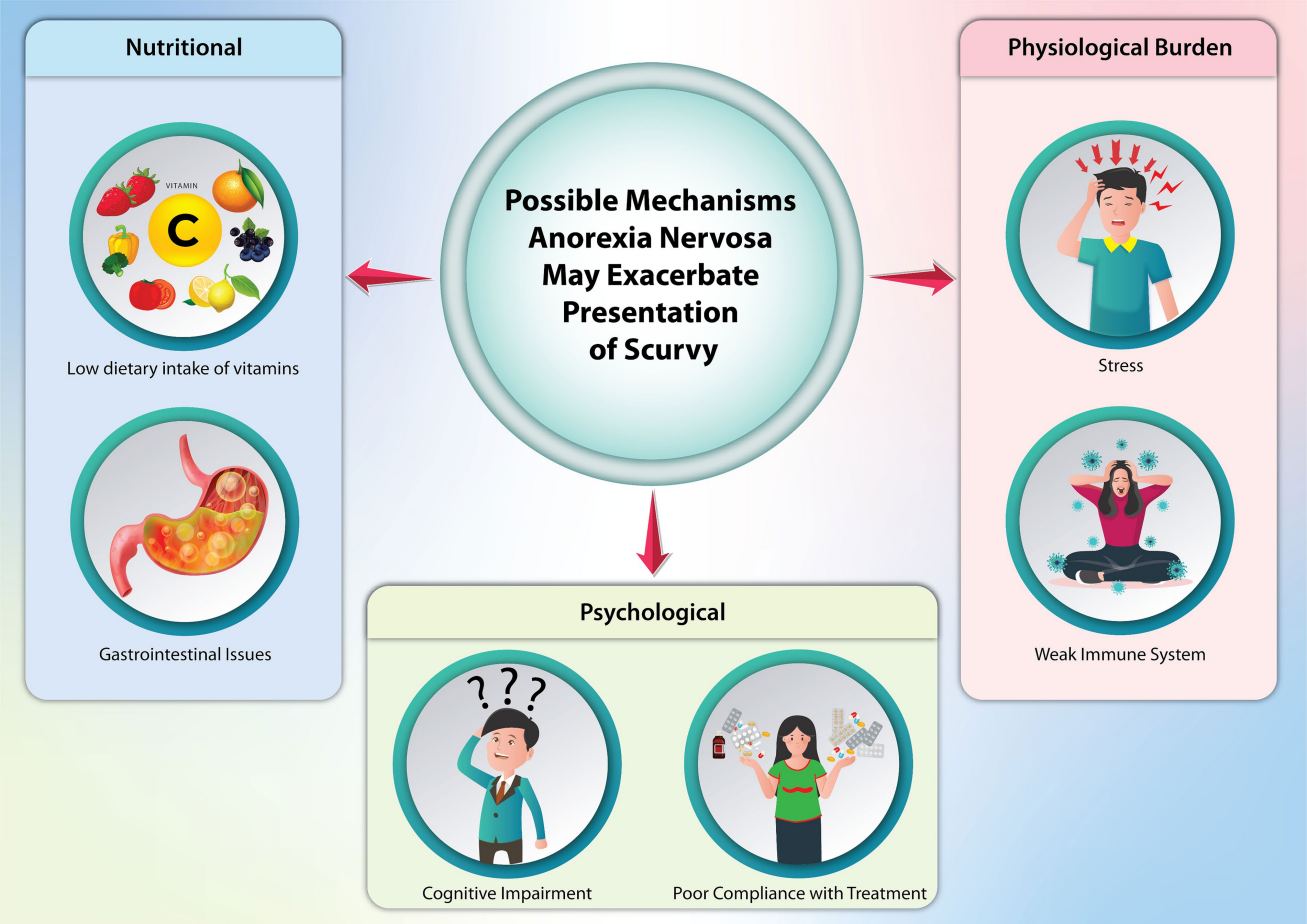
Possible mechanisms anorexia nervosa may exacerbate the presentation of scurvy.

AN can alter the presentation of symptoms traditionally associated with scurvy, given the widespread physiological impacts of AN on various body systems (Figure 3) (13). It is reasonable to believe a patient with AN can eventually develop scurvy due to malnutrition. AN-related conditions like gastrointestinal distress, reduced appetite, and impaired nutrient absorption (such as vomiting and diarrhea) can deplete essential vitamins and worsen or exacerbate the overall nutritional status of a patient. AN can also obscure the presence of scurvy. For instance, fatigue, malaise, and dermatological changes are characteristic of both, and providers may potentially focus on AN treatment with little avail if vitamin deficiencies are not addressed first.

Despite being an ancient disease, scurvy remains prevalent. This is particularly true among individuals with restrictive eating disorders and other at-risk populations (28). Modern cases often arise from inadequate dietary intake, a situation not uncommon even in developed societies. Scurvy’s clinical presentation can also be mistaken for other conditions, such as rheumatoid arthritis, clotting disorders, or anemia, due to symptoms like joint pain, petechiae, and anemia (29). Additionally, other vitamin deficiencies, such as beriberi (vitamin B1 deficiency) and pellagra (vitamin B3 deficiency), can present with neuropsychiatric and dermatological symptoms similar to those of scurvy and also co-present with AN, further complicating the clinical picture (30, 31).

The complexity of treating these overlapping conditions is heightened by the fact that anorexia nervosa, a psychiatric illness characterized by a refusal to consume food, inherently leads to macro and micronutrient deficiencies. This refusal, along with other hallmark features of AN such as distorted body image and an intense fear of gaining weight, directly impacts patients’ adherence to treatment regimens. Moreover, the stigma and psychological burden of eating disorders can lead to underreporting of symptoms, further delaying accurate diagnosis.

Given the common symptoms, healthcare providers should likely maintain suspicion for vitamin deficiencies in patients with restrictive eating behaviors. Nonetheless, AN and scurvy can differ in crucial ways, and differential diagnosis is possible. Specifically, petechiae, purpura, and perifollicular hemorrhages may more likely indicate scurvy in patients with AN, as these symptoms are not typically observed in AN alone. Supporting this, all nine case reports in this study documented such symptoms in their patients, all occurring within lower extremities. This is likely due to increased hydrostatic pressure caused by gravity, which exacerbates the fragility of blood vessels weakened by vitamin C deficiency, resulting in subcutaneous internal bleeding. Symptoms of gum disease may also assist in diagnosis, with this study finding three cases (18, 19, 23) reporting gingival bleeding or irritation in their patients. Additionally, corkscrew hairs can serve as a strong indicator to parse out AN and scurvy, as vitamin C deficiency reduces disulfide bonds and keratin cross-linkage in hair (32). As evidence of this, three cases (19, 21, 33) in this study report such symptoms. However, corkscrew hairs are not invariably present in patients with scurvy, as they present in typically older patients and may not be obvious if the period of vitamin deficiency does not coincide with active hair growth phases (32). While delayed wound healing may not be immediately apparent upon a patient’s hospital admission and was not reported in any case within this study, it can also serve as a differential indicator (34). Finally, anemia was reported in one patient (22). Vitamin C enhances absorption of non-heme iron and reduces ferrous iron to ferric iron, and thus scurvy patients are often reported to have microcytic anemia (35). In contrast, microcytic anemia is rare in AN, further suggesting the need to evaluate for concomitant vitamin C deficiency when such anemia is observed. However, other forms of anemia from vitamin deficiencies can present similarly, such as megaloblastic anemia due to folate or vitamin B12 deficiency, or hemolytic anemia due to vitamin E deficiency (36, 37). Thus, a combination of indicators and symptoms should be used by clinicians to accurately diagnose patients who may have scurvy co-presenting with AN, allowing for earlier further evaluation of vitamin C deficiency in hospital settings.

One limitation of this review is the limited number and diversity of case reports available on the concurrent presentation of scurvy and AN. Due to the rarity of these comorbid conditions, detailed literature on patient demographics, symptoms, and treatment regimens is scarce. Most existing reports focus on female patients, even though men are estimated to account for approximately 25% of all AN cases (38). This lack of male representation could lead to an incomplete understanding of how scurvy manifests in male patients with AN, given potential biological differences in nutritional needs and metabolic responses. Consequently, the current literature may not fully capture the variability in symptom presentation and treatment outcomes across different genders. Furthermore, the retrospective nature of this study introduces inherent limitations, such as the inability to control for confounding variables and biases present in the original reports. Examples of confounding variables include the severity and duration of AN, variations in patients’ overall nutritional status, and the presence of other comorbid conditions such as depression, anxiety, or gastrointestinal disorders that can affect nutrient absorption and overall health. Additionally, differing healthcare access and treatment adherence among patients can further complicate the interpretation of case reports. Publication bias is another significant concern, as more severe or unusual cases are more likely to be reported, potentially skewing the overall understanding of these comorbid conditions’ typical presentation and progression. This bias may lead to an overemphasis on atypical cases, making it difficult to generalize findings to the broader population of patients with both scurvy and AN. Future research should aim to include a wider range of case reports and prospective studies to provide a more comprehensive and balanced understanding of these conditions.

In conclusion, the intersection of scurvy and AN presents a complex clinical challenge, highlighting the significant impact of vitamin C deficiencies in AN patients. Recognizing the distinct systemic presentations of AN and scurvy is essential due to overlapping symptoms such as fatigue, malaise, and dermatological changes. Differentiating scurvy from AN involves identifying symptoms that are uncommon in AN alone. Petechiae, purpura, and perifollicular hemorrhages, especially in the lower extremities due to weakened blood vessel walls, are strong indicators of scurvy. Gingival bleeding, corkscrew hairs, and anemia can further aid in distinguishing between the two conditions. These clinical signs, when present, should prompt consideration of scurvy in AN patients and lead to further diagnostic measures such as a plasma ascorbic acid test. A comprehensive treatment approach involving immediate vitamin C supplementation, increased dietary intake, and ongoing psychiatric support is essential. Furthermore, more comprehensive nutritional assessments may improve early detection and subsequence management, potentially mitigating severe complications. This review highlights the limitations in the current literature, as few detailed case reports on concurrent scurvy and AN exist. This is particularly true for male patients, resulting in an incomplete understanding of these comorbid conditions. Future research should include diverse case reports and prospective studies, considering biological differences between genders and addressing psychological factors that hinder treatment adherence. Furthermore, given the small sample size in the current review, a larger controlled study in a representative sample is necessary to accurately identify the prevalence of scurvy in the population. It is also essential clinicians remain vigilant about the possibility of specific vitamin C deficiency in patients with AN, allowing for early detection and timely treatment of scurvy.

## Data Availability

The original contributions presented in the study are included in the article/supplementary material, further inquiries can be directed to the corresponding author.
